# The Medical Schools of London

**Published:** 1889-09-14

**Authors:** 


### The Medical Schools of London.

ARRANGEMENTS FOR THE SESSION 1889—90, WITH THE SPECIAL FEATURES AND COST OF CURRICULUM AT EACH INSTITUTION.

### Charing Cross Medical School.

The winter session will commence on October 1, but no inaugural address will be given.

The following changes have taken place in the staff of the school during the year: Mr. Marmaduke Sheild has been appointed to the new post of surgical tutor; Dr. Amand Routh has been appointed teacher of practical obstetrics and gynæcology; Messrs. F. C. Wallis and C. J. Woollett have been appointed demonstrators of anatomy; Mr. Stanley Boyd has been appointed teacher of operative surgery.

The following scholarships are open to students at Charing Cross Hospital: two entrance scholarships, of the value of 100 guineas and 50 guineas respectively, and a scholarship of the value of 50 guineas.

The fee for lectures and hospital practice is £94 10s.; or in instalments of £30 9s., £18 18s., £17 17s., £18 18s., and £18 18s.

The medical school is being considerably enlarged, and, in addition to other improvements, new physiological and pathological laboratories will be erected, and an additional reading and writing room provided for the use of the students.

### Guy's Hospital Medical School.

The session of 1889-90 commences on Tuesday, October 1, 1889. The first meeting for the session of the Physical Society will be held on the evening of the same day in the Anatomical Theatre, at eight o'clock. The chair will be taken by Dr. Wilks, F.R.S. A. G. Barrs, Esq., M.D., will read a paper on "Some Modern Tendencies in Medical Education." The rooms of the Students' Club will be open from 7-8 p.m. before the meeting for the exhibition of instruments, microscopical preparations, and photographs taken by members of the society during the past year. All past and present students are invited to attend.

The following scholarships are open to students at Guy's Hospital:

Entrance Scholarships in Arts.—(1) A scholarship of 100 guineas, open to candidates under 20 years of age; (2) a scholarship of 50 guineas, open to candidates under 25 years of age. Entrance Scholarships in Science.—(1) A scholarship of 125 guineas, open to candidates under 25 years of age; (2) a scholarship of 50 guineas, open to candidates under 25 years of age. The Arthur Durham Prizes for Dissection.—One of £5 to students in their first year, the other of £15 to students of later years.

For students in their first year: First prize, £50; second prize, £25. For students in their second year: First prize, £25; second prize, £10. Michael Harris prize, of the value of £10. For students in their third year: First prize, £25; second prize, £10. For students in their fourth year: First prize, £25; second prize, £10. Golding Bird prize, consisting of a gold medal of the value of £13, and a sum of money of about £20.

For students in their fourth or fifth year: The Treasurer's Gold Medal for Clinical Medicine. The Treasurer's Gold Medal for Clinical Surgery. Gurney Hoare prize, of the value of £25. The Beaney prize, of the value of £31 10s.

Fee for lectures and hospital practice: By the payment of 125 guineas on entrance; or by two payments of £66; or by three annual instalments of £50, £50, and £37 10s.

A residential college is being erected, which is connected with the hospital by a subway, and contains accommodation for fifty students, in addition to the resident medical officers of the hospital. The terms vary from 27s. to 10s. a week, according to the accommodation afforded. The college will be opened about Christmas.

A dental school has recently been inaugurated in connection with the hospital.

### King's College Hospital Medical School.

The session commences on Oct. 1st. The following changes have taken place during the year: Mr. Henry Smith has been made consulting surgeon, and Emeritus Professor of Surgery. Mr. Rose has been appointed surgeon. A chair of Neuro-pathology has been instituted, and Dr. Ferrier has been appointed to it. Dr. W. R. Smith has been appointed to the chair of forensic medicine, vacated by Dr. Ferrier. Mr. Carless has been appointed assistant surgeon, and assists Mr. Barrow in the course on practical surgery. Mr. A. S. Underwood has been appointed dental surgeon. Dr. Dalton lectures on pathology and morbid anatomy, and is also demonstrator of morbid histology and practical pathology. Mr. W. K. Dale has been appointed demonstrator of bacteriology.

The following scholarships, etc., are open to students:—Five Warneford Scholarships of £25 per annum, tenable for three years; a Sambrooke Exhibition of £60 and one of £40; Rabbeth Scholarship of £20; a Science Exhibition of £50 per annum and one of £25 per annum, each tenable for two years; Medical Scholarships of £40, £30, and £20; two Sambrooke Registrarships of £50; Daniel Scholarship of £20 per annum for two years.

Fee for lectures and hospital practice, £126 11s. 6d.; or in two annual instalments of £66, or three of £46 each.

### London Hospital Medical School.

The winter session commences on October 1. No inaugural address will be given, but the "old students dinner" will be held in the library of the college in the evening, Dr. Langdon Down, senior physician to the hospital, presiding. During the year Mr. John Cowper, the senior surgeon, has retired, his full term of service having expired. Dr. Prosser James, the lecturer on Materia Medica, has also resigned, but no new appointments have yet been made in either case.

The following scholarships, etc., are open to students of the London Hospital: Two entrance scholarships of £60 and £40 respectively; a Buxton Scholarship of £30, and four of £20; a Duckworth-Nelson Prize of £10; three Letheby Prizes of £30, £25, and £20; two Dressers' Prizes of £15, and two of £10.

Fee for lectures and hospital practice, £94 12s.; or £105 in three instalments.

Students wishing to reside with a member of the medical profession living within easy access to the hospital can obtain information as to vacancies and terms by application to the warden, either personally or by letter. Several members of the hospital staff receive resident pupils. Lodgings can be obtained in quiet and healthy localities in the immediate vicinity of the hospital, or in the neighbourhood of Victoria Park, at very reasonable charges. A list of approved lodgings is kept by the warden. A dining and refreshment room for the use of students is provided in the building, so that luncheons or dinners may be obtained without the necessity of quitting the precincts of the college.

### Middlesex Hospital Medical School.

The session begins on October 1, 1889, when the inaugural address will be given by Mr. Bland Sutton. After the address, Lord Sandhurst will distribute the prizes to those students who have distinguished themselves during the past year. This will be followed by a reception at the college at 4.30. The following changes have occurred in the staff during the last year: Dr. Greenhow, consulting physician, has died; and Dr. Edis has resigned. The following appointments have been made: Dr. W. Duncan, obstetric physician, and lecturer on midwifery; Dr. R. Boxall, assistant obstetric physician, and lecturer on practical midwifery; Dr. W. Pasteur, assistant physician, with special charge of the children's department; Dr. W. E. Wynter, medical registrar; and Mr. T. Carwardine, junior demonstrator in anatomy.

The following scholarships are open to students at Middlesex Hospital: Two entrance scholarships of £50 and £30, respectively, tenable for two years; two Broderip Scholarships of £30 and £20, respectively, tenable for two years; obtained by success in an examination on clinical subjects; Governors' Prize of £21, open to third year's students, obtained by success in an examination on clinical subjects.

Fee for lectures and hospital practice, £100.

A residential college for students is attached to the hospital. There is a cancer ward in the hospital, containing thirty-four beds, and a children's department has recently been established. A new post-mortem room and mortuary are almost completed. The site of the present post-mortem room will be occupied by a chapel.

All information can be obtained from the dean, Mr. A. Pearce-Gould.

### St. Bartholomew's Hospital Medical School.

The winter session opens on 1st October, 1889, but no inaugural address will be given. The following changes have taken place in the staff during the last year: Mr. Willett and Mr. Marsh have been appointed lecturers on surgery, in succession to Mr. Savory; Mr. Walsham and Mr. Bruce Clarke, lecturers on anatomy, in succession to Mr. Langton and Mr. Marsh; Mr. Bowlby and Mr. D'Arcy Power, demonstrators in surgery; Dr. Rolleston and Mr. Bulgarnie assistant demonstrators in anatomy; and Dr. Hamer assistant demonstrator in physiology.

The following scholarships are open to students of St. Bartholomew's: One entrance scholarship of £130, and two of £65; Preliminary Scientific Exhibition of £50; Jeaffreson Exhibition (for proficiency in arts) of £20; Shuter Scholarship of £50; two Junior Scholarships of £40 and £20 respectively; and one Senior Scholarship of £50; Kirkes' Scholarship of £30 and Medal; and two Brackenbury Scholarships of £30 each; Lawrence Scholarship of £42 and Medal; Sir George Burrows' Prize (£10 10s.), for Pathology; and Skynner Prize of £15 for Morbid Anatomy.

Fee for lectures and hospital practice: £131 5s.; or in instalments of £42, £48 6s., and £48 6s.

A resident college for students is attached. N. Moore, M.D., warden.

### St. George's Medical School.

The winter session will commence on Tuesday, October 1, with an introductory address by Dr. T. Clifford Allbutt, at 4 p.m.

The following changes have taken place during the last year: Dr. Penrose has been elected assistant physician in succession to Dr. Gamgee, and Mr. Lancaster has been appointed demonstrator in anatomy.

The following prizes are open to students at St. George's Hospital: Five entrance scholarships of the value of £125, £90, £65, £50, and £50 respectively; two Wm. Brown Exhibitions, one of £100 per annum tenable for two years, and one of £40 tenable for three years; a Brackenbury Prize in Medicine and one in Surgery, consisting in each case of the interest on £1,877; a Treasurer's Prize of £10 10s.; an H. C. Johnson Memorial Prize of £10 10s.; and a Pollock Prize of the interest on £372; besides three General Proficiency Prizes of £10 10s. each. The Thompson Medal, consisting of the interest on £171 1s. 11d. Sir Benjamin Brodie's Clinical Prize in Surgery, consisting of the interest on £207 19s. 7d. The Acland Clinical Prize in Medicine, value £5.

Fee for lecture and hospital practice, £125; or in three instalments of £48 13s. 6d., £48 13s. 6d., and £40 10s. 6d.

Adjoining the school, but not under the same roof—an obvious advantage from a sanitary point of view—is the students' club, which includes a buffet and coffee-room, where wholesome meals may be obtained at a moderate and fixed rate; leading from this is the reading-room, which is supplied with daily papers, stationery, etc.

Forms for registration may be obtained from the dean, or by application at 299, Oxford-street.

### St. Mary's Hospital Medical School.

The session begins on Tuesday, October 1, when Dr. Maguire will give the introductory lecture. The following changes have taken place during the last year: Dr. Thomas King Chambers, senior consulting physician, has died; Dr. Phillips and Mr. Owen have been appointed physician and surgeon, respectively, to the new out-patient department for children; Mr. J. J. Clark has been appointed curator of the museum, and Mr. Roughton a senior demonstrator of anatomy; Mr. H. W. Page has been appointed dean of the school in place of Mr. Field, who has resigned.

The following scholarships are open to students at St. Mary's Hospital: A Natural Science Scholarship of £105, and three ditto of £52 10s. each; a prize of £105 obtainable by students from Epsom College who are sons of medical men; two prizes of £52 10s. each for success in Preliminary Scientific Examination of London University, and prizes of £50, £50, £30, £25, and £20 respectively.

Fee for lectures and hospital practice, £100; or in three instalments of £40, £40, and £25.

### St. Thomas's Hospital Medical School.

The winter session 1889-90 will commence on Tuesday, October 1, and terminate on March 31. The summer session will begin on May 1, and terminate on July 31. An introductory address will be given in one of the theatres of the hospital by William Anderson, Esq., F.R.C.S., on Tuesday, October 1, at 3 p.m., after which the various departments of the hospital and school will be thrown open in working order for the inspection of visitors. Refreshments will be provided in the library. The annual dinner, in which all former and present students are invited to join, will take place the same evening in the governors' hall at 6 for 6.30, John Harley, M.D., in the chair.

The annual distribution of prizes will be made during the summer session.

The following scholarship, prizes, and medals will be offered for competition during the year 1889-1890: Two open scholarships in natural science of the value of 125 guineas and £60, respectively, at entrance. At the end of first year.—Winter: 1, the William Tite Scholarship, £27 10s.; 2, college prize, £20; 3, ditto, £10. Summer: 1, college prize, £15; 2, ditto, £10. Second year.—Winter: 1, the Peacock Scholarship, £38 10s.; 2, college prize, £20; 3, ditto, £10. Summer: 1, college prize, £15; 2, ditto, £10. Third Year.—Winter: Second tenure of the Musgrove Scholarship (if holder obtains 1st class in this examination, £38 10s.; 1, college prize, £20; 2, ditto, £15; 3, ditto, £10. Summer: 1, college prize, £15; 2, ditto, £10.

Free scholarships are given to distinguished pupils of Merchant Taylors' and City of London Schools, and Epsom College. In addition there are awarded: The Cheselden Medal, annually; the Mead Medal, annually; the Solly Medal and Prize, biennially; the Grainger Testimonial Prize, annually; the Treasurer's Gold Medal, annually.

The following changes have taken place in the staff of the hospital. Dr. Gulliver gives demonstrations in morbid anatomy, in addition to Drs. Sharkey and Hadden; Mr. Parsons has been appointed junior demonstrator of anatomy, and Mr. Sydney Plowman gives up the joint lectureship in Materia Medica and his post as apothecary to the hospital, in order to fill the appointment of director of the College of Pharmacy, Melbourne, and demonstrator of pharmacy in the University of Melbourne.

A register of lodgings suitable for students has been recently revised, and is kept in the secretary's office. Information as to terms, accommodation, etc., can be obtained on application. This register has been especially prepared with a view to the convenience of new students, for whose accommodation in lodgings or otherwise no definite arrangements have been made. Medical practitioners, clergymen, and private families residing in the neighbourhood receive students for residence and supervision.

Students' Club.—For the convenience of students, a club has been established in the medical school building. The club premises comprise a dining-room, where between 9 a.m. and 6 p.m. refreshments can be obtained at reasonable prices; and a reading and smoking room supplied with most of the daily and illustrated weekly papers. There is no annual subscription, but students who join in their first year pay £1 1s. for permanent membership; those joining in their second year, 10s. 6d.; and those of later years, 5s. The fees are received by the secretary of the club, Mr. G. S. Saunders, librarian. The club can be used only by members.

Fee for lectures and hospital practice, £125; or by two instalments, £75 and £63; or by three instalments, £65, £50, and £30.

For information on all matters relating to the medical school, prizes, scholarships, etc., application should be made to the medical secretary, Mr. G. Rendle, at the hospital, Albert-embankment, S.E., personally (10 to 4, Saturday 10 to 1), or by letter.

### University College Medical School.

Dean.—Professor John Williams, M.D. Vice-Dean.—Professor E. A. Schäfer, F.R.S. Sub-Dean.—Professor G. D. Thane.

The session will be opened on Tuesday, October 1, at 4 p.m., when an introductory address will be delivered in the Botanical Theatre by Mr. R. J. Godlee, M.S., B.A., F.R.C.S., surgeon to University College Hospital. The annual dinner of the old and present students of the Faculty of Medicine will be held in the General Library of University College on Tuesday, October 1, 1889, at 6.30 p.m. William Cadge, Esq., F.R.C.S., member of the Council of the Royal College of Surgeons, surgeon to the Norfolk and Norwich Hospital, will preside.

The dean, vice-dean, and sub-dean will always be ready to give information and advice to students or to their friends; they will attend specially for this purpose on September 30 and October 1, from 2 to 4 p.m., and on April 30 and May 1, at the same hours; and students joining the classes of the faculty for the first time are urged to consult them on their course of study. The sub-dean may also be seen daily (except Saturday) from 1 to 2 p.m., and at other times by appointment. Parents or guardians requiring information about students should apply, either personally or by letter, to the dean or sub-dean. Students who, from illness or other cause, are absent from the college for more than three consecutive days, are required to communicate with the sub-dean, and inform him of the reason of their absence. A register of students' addresses is kept at the office of the college, and it is requested that early information be given of any change of residence.

Exhibitions.—Exhibitions and prizes of the annual value of about £640 are open to competition among students of the Faculty of Medicine, and in addition sixteen gold medals and thirty silver medals may be awarded annually.

Entrance Exhibitions.—The Council of University College will award exhibitions of £100, £60, and £40 respectively every year for proficiency in science: the subjects of examination being those of the Preliminary Scientific Examination of the University of London.

Other prizes are: the Atkinson Morley Surgical Scholarships, amounting to £45 per annum, tenable for three years; an Atchison Scholarship, value about £60 per annum, tenable for two years; a Sharpey Physiological Scholarship, about £105; Filliter Exhibition of £30; Erichsen Prize, a surgeon's operating case, of the value of ten guineas; and Morris Bursary, of £25 a year, for sons of deceased professional men.

Prizes to the value of £10 will be given in the class of hygiene, on conditions stated in the programme of the class.

In the anatomical department a much-needed improvement has been effected by the reconstruction and extension of the northern portion of the block hitherto known as the small dissecting-room. The new building, which is in two storeys, contains a small theatre for demonstrations, a commodious operative surgery-room, a preparation-room, and two work and store rooms for the professor and his assistants. Additional lavatory accommodation for the students has also been provided, and the professor's study has been enlarged. The rooms are well lighted, and are fitted up with all necessary conveniences, including work-tables and sinks, with hot and cold water supply, gas, and hot-water warming apparatus. There is a lift for the conveyance of heavy objects between the two floors. The alterations as carried out have met with general approval, and constitute a valuable addition to the medical school.

Fee for lectures and hospital practice, £126; or by instalments of £57 15s., £52 10s., and £21.

### Westminster Hospital Medical School.

The session begins on Wednesday, October 2, when Dr. P. S. Abraham will deliver the inaugural lecture. During the year the following changes have taken place in the hospital staff: Dr. C. B. Radcliffe, consulting physician, has died; Dr. Hebb has been appointed assistant physician; Dr. Mercier succeeds Dr. Sutherland as lecturer on mental diseases. In the course on practical surgery, Mr. Davy will be assisted by Messrs. Stonham and Spencer.

The following scholarships are open to students of the Westminster Hospital: A Natural Science Entrance Scholarship of £100; two entrance scholarships of £40, two of £20, and two of £10; a Guthrie Scholarship of £40; a Rutherford Alcock Scholarship of £40; two Senior Scholarships of £20 (all the above are tenable for two years); a Treasurer's Prize of £10 10s.; a President's Prize of £21; and a Chadwick Prize of books or instruments to the value of £20.

Fee for lectures and hospital practice, £105; or two instalments of £55; or five instalments of £24.

